# Facilitation of Evolution by Plasticity Scales with Phenotypic Complexity

**DOI:** 10.3390/ani14192804

**Published:** 2024-09-28

**Authors:** Mikhail Burtsev, Konstantin Anokhin, Patrick Bateson

**Affiliations:** 1 London Institute for Mathematical Sciences, Royal Institution, London W1S 4BS, UK; 2 Institute for Advanced Brain Studies, Moscow State University, Moscow 119992, Russia; 3 Sub-Department of Animal Behaviour, University of Cambridge, Cambridge CB28 3AA, UK

**Keywords:** developmental plasticity, learning, evolutionary biology, genetic assimilation

## Abstract

**Simple Summary:**

Developmental plasticity allows organisms to adapt quickly by altering their behaviour or physiology, often at a high energy or time cost. This flexibility can lead to more permanent genetic changes, simplifying the organism’s response to similar future challenges. Our research shows that plasticity not only speeds up the evolution of complex behaviours in organisms but also plays a crucial role in the development of increasingly complex biological systems. As organisms face more difficult environmental tasks, plasticity becomes a more powerful tool in facilitating rapid evolutionary advancements and the diversification of species.

**Abstract:**

Developmental plasticity enables organisms to cope with new environmental challenges. If deploying such plasticity is costly in terms of time or energy, the same adaptive behaviour could subsequently evolve through piecemeal genomic reorganisation that replaces the requirement to acquire that adaptation by individual plasticity. Here, we report a new dimension to the way in which plasticity can drive evolutionary change, leading to an ever-greater complexity in biological organisation. Plasticity dramatically accelerates the evolutionary accumulation of adaptive systems in model organisms with relatively low rates of mutation. The effect of plasticity on the evolutionary growth of complexity is even greater when the number of elements needed to construct a functional system is increased. These results suggest that, as the difficulty of challenges from the environment becomes greater, plasticity exerts an ever more powerful role in meeting those challenges and in opening up new avenues for the subsequent evolution of complex adaptations.

## 1. Introduction

The role of plasticity in the development and evolution of both plants and animals is attracting a great deal of current interest [1,2,3,4,5,6,7,8]. Ideas about the role of plasticity in evolution have had a long history. The most famous hypothesis is thought to have originated with Baldwin [9], Lloyd Morgan [10], and Osborne [11], who independently suggested that an individual’s adaptability could prepare the ground for an evolutionary change in which the same outcome is eventually achieved without such plasticity. However, the idea was originally proposed 23 years earlier by Spalding [12]). Bateson [13] has suggested, therefore, that the misleading term “Baldwin effect” should be replaced by the descriptive term “Adaptability driver”. The mechanism for how plasticity could influence evolution has intrigued many authors [8,14,15,16,17,18,19,20,21,22,23]. West–Eberhard [24] has been a prominent advocate of the view that an individual’s plasticity plays an important role in evolution, and she has been supported by other influential writers [25].

The hypothesis that adaptability drives evolution has been repeatedly modeled both analytically [25,26,27,28,29,30,31] and by simulation [32,33,34,35,36,37]. Nevertheless, the hypothesis is widely supposed to be of limited interest because it merely proposes a mechanism by which one phenotype acquired through the organism’s adaptability is replaced, in the course of Darwinian evolution, by an inherited mechanism that expresses itself as a phenotypic copy at a lower cost. The hypothesis was not thought to provide a general explanation for evolutionary processes that result in the accumulation of phenotypic complexity [38].

The standard explanation given for the evolution of complex phenotypic traits, including adaptive behaviour, is that it involves the gradual accumulation of the necessary component processes by reorganisation of the genome alone [39,40]. The problem is that, in most cases, even the evolution of the simplest adaptive trait requires a number of independent reorganisations of the genome, including multiple gene mutations, changes in gene regulation, chromosomal rearrangements, and alterations in genetic networks that collectively contribute to the development and expression of the new trait. Evolutionary and developmental interactions between genotype and phenotype lead to highly nonlinear dynamics of evolvability [41,42,43,44,45,46,47,48,49,50]. When the adaptation depended on the simultaneous occurrence of a combination of such reorganisations that would be useless on their own, inevitably the evolutionary process would have been slow. Moreover, the time to acquire the adaptation by mutation grows nonlinearly with the required number of genomic reorganisations. Suppose that every reorganisation is expected to appear in a population every 10 generations; then, the addition of one more reorganisation to the combination will delay the appearance of the adaptation 10-fold. Another complication arises from the fact that, due to pleiotropy, a single mutation can affect multiple phenotypic traits. Yet, a growing body of evidence suggests that complex behaviour has evolved rapidly in birds and mammals [51,52,53,54]. In this paper, we show how such integrated systems could appear rapidly in the course of evolution under the guidance of an individual’s plasticity and the use of elements shared by different functional systems. Many different forms of plasticity have been recognized, including the various forms of learning operating at the behavioural level, while other forms of plasticity operate at lower levels of organisation [3]. In this study, we consider plasticity as the functional capacity of an individual to adapt its phenotype for environmental challenges through non-heritable modifications.

We argue that the effect of plasticity on evolution became increasingly powerful as animals became more complex. As components of functional systems requiring plasticity are genetically assimilated in the course of evolution, then less and less plasticity is required to integrate inherited elements into the functional system. If the capacity for plastic change remains constant, it can be used by an animal to acquire other, previously unavailable, adaptations. This “assimilate-stretch” process [22] creates constant pressure for further assimilation and for retaining plasticity. In our study, we examine how such a process guided by plasticity and relying on partial overlap between different functional systems leads to the accumulation of complexity in biological organisation.

## 2. Materials and Methods

We incorporated into our simulation the concept of “functional systems” [55]. The general idea is that the fitness of an individual depends on systems organised as different combinations of phenotypic elements such as connections in the neural network, the capacity to use information contained in the energy impinging on a sense organ, specific biochemical reactions, and particular effectors that respond adaptively to the stimulation. Elements may be recombined in different ways to perform different functions. Novel challenges create the conditions for the emergence of new functional systems added to the existing ones either by Darwinian evolution or by an individual’s plasticity. Possible examples are the addition of a face recognition module in primates [56] and the evolution of habitat invasiveness in birds [52]. This evolutionary process leads to the establishment of an increasingly elaborate phenotypic organisation and patterns of behaviour. When such complexity entails a greater ability to discriminate between different features of the environment or a greater ability to manipulate the environment, the organism will benefit and will be more likely to survive and reproduce in the face of multiple challenges during its lifetime.

A new inherited adaptation emerges in evolution when the accumulated phenotypic effects of genomic reorganisation are added to the existing phenotype. Although these phenotypic effects are specific to the new function, existing parts of the phenotype are also recruited for this function. As a result, phenotypic elements established earlier in evolution should be incorporated into more adaptive systems than the later-evolved elements. To simulate this process, the functional systems in our model were nested in a hierarchy from old to new. In evolution, this hierarchy forms a structure similar to a tree but with the possibility that some existing branches may be recombined later in evolution to form new functional. The goal of our study is to show how plasticity can accelerate the growth of this phenotypic hierarchy. To make our results clear, we focused on the growth along a single line of branches. However, our results can be easily generalized to the full hierarchy by assuming that multiple lines grow in parallel.

**Figure 1:**
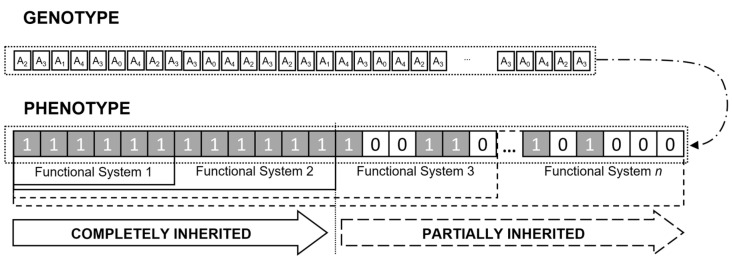
The model of an organism capable of plasticity. The phenotype of an individual in our model developed from a set of genes through a genotype–phenotype map, forming a hierarchy of nested functional systems. Each functional system consisted of phenotypic elements, the number of which is specified by a parameter *C* (*C* = 6 for the phenotype on the diagram). An element is either present (‘1’) or absent (‘0’). Each inherited element is influenced by a gene. An absent element may be made operational (‘0’ converted to ‘1’) by genetic mutation or by plasticity. In each individual model, organism development proceeded step by step through fully inherited systems until a system was reached in which an element was missing. In members of the populations that exhibited plasticity, an element could be made operational by processes that replaced a ‘0’ with a ‘1’ over a fixed number of trials specified by a parameter (*t*). The organisms with the largest number of complete functional systems are most likely to survive and reproduce (the phenotype on the diagram has a fitness equal to two).

The model organism in our simulations was made up of functional systems, and each system consisted of a number of elements (Figure 1). The phenotype is described by the set **P** of *f* binary strings given by **e***^i^* for the *i*th functional system:

(1) $$P=\left\{e^{i}:i\in \left[1;f\right]\right\},$$

where *f* is the maximal possible number of functional systems per organism during the simulation. Components of the given binary string encoded the presence of the given feature in the phenotype. If the value of a component element was 1, then the corresponding feature was included in the phenotype, otherwise it was not. The length of the string is given by the parameter *C*, which defines phenotypic complexity as a number of elements required to achieve an adaptive function. A new system was composed by the addition of *C* elements to the terminal system; thus, the functional system **FS_j_** included all strings **e***^i^* with *i* ≤ *j*:

(2) $${FS}_{j}=\overset{j}{\underset{i=1}{⋃}}e^{i},$$

where ∪ is a union operator representing the concatenation of binary strings with indexes from 1 to *j* to form a representation of the *j*th functional system in the model. Each element was necessary for the working of the whole functional system, and the system would not become functional until it had a complete set of elements, i.e., all their bits were set to 1. Only completed systems contributed to the organism’s fitness.

The fitness of the phenotype was equal to the number of complete systems. In the real world, the complexity of an animal can decrease in the course of evolution, as in the case of many parasites, but in the cases we sought to model, the capacity to handle a large number of different challenges by the environment carried a distinct advantage.

The genotype of the individual consisted of *L* loci with *A* alleles per locus. Every allele was connected to some phenotypic element, and if this allele was expressed in the genotype, then the corresponding element was manifested in the phenotype. Thus, the genetic architecture of each individual specified the inherited states of each of its system’s elements.

The parent’s genome was inherited by the offspring, so their phenotypes were the same, except that each element could be changed by a point mutation in a gene relating to that element. Genomic reorganisation could add or remove an element from a functional system. For simplicity, we have represented such reorganisation as a single point mutation.

An individual in the model was initialised by the sequential assembling of the inherited systems from a genome, guided with genotype–phenotype map. This development terminated when the first system with a missing element was encountered. If, due to mutation, some phenotypic element of an intermediate system was lost, then the developmental progression was broken at this stage, and none of the functional systems at subsequent developmental stages were able to make contributions to fitness. On the other hand, a mutation could produce an element that completed a new terminal system and increased the organism’s fitness. The difficulty of acquiring a new system was affected by the parameter *C* that determined the number of elements required for a complete system.

A new system could be also generated within an individual in those populations in which plasticity was possible. Plasticity can switch the states of phenotypic elements from 0 to 1. During the period of plasticity, an individual was allowed to perform a fixed number of attempts to fill in independently, with probability *p_e_*, the missing phenotypic elements in the terminal system. Thus, in our model, plasticity was guided by evolved developmental predispositions within partially inherited functional systems, operating as an augmentation of existing systems rather than acting on a random phenotype. Given *t* trials, an individual was able to complete one system per trial with the following probability:

(3) $$p_{FS}=p_{e}^{m},$$

where *m* is the number of missed elements in the system *FS*. Hence, the more elements that were missing, the less likely it was that plasticity would be successful. If the trial was successful, the remaining trials were utilized for further development by the probabilistic addition of subsequent functional systems. Acquired elements were not inherited; Lamarckian inheritance did not occur in this model. However, later in evolution, the element filled by plasticity might be replaced by genomic reorganisation, simulating the adaptability driver. In our model, the replacement by the genomic reorganisation of an element previously filled by plasticity released the descendents of that organism to meet other challenges set by the environment.

When the phenotype of each individual in the population were specified, *N* offspring that would constitute the next generation were obtained. A potential candidate for breeding was selected at random from the population. The probability *p_rep_* that it would reproduce was equal to its number of completed functional systems relative to the individual in the population with the largest number of functional systems:

(4) $$p_{rep}^{i}=\frac{n_{FS}^{i}}{\underset{N}{\max}\left(n_{FS}^{j}\right)}\;,$$

where $n_{FS}^{i}$ is a number of completed systems for *i*th individual and *N* is a population size. The offspring had the same genotype as the parent before point mutation was applied to each locus with probability *µ*. A summary of the simulation routine is given in Algorithm 1.
**Algorithm 1.** Plasticity guided evolution
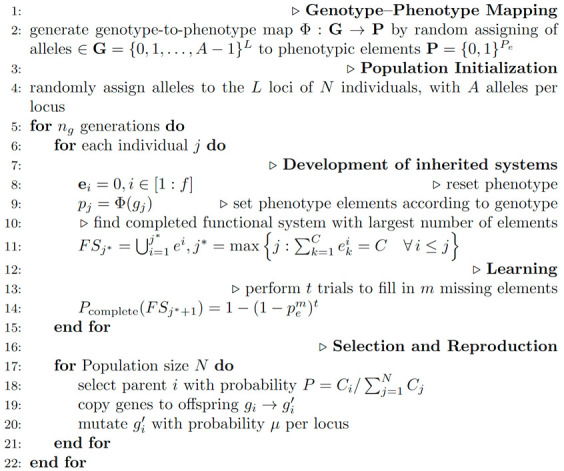


## 3. Results

In each simulation, a population of *N =* 500 individuals was established, and the alleles of individual’s genotypes were randomly set. By the genotype–phenotype map the genetic architecture of each individual specifies the states of each of its systems’ phenotypic elements. In our experiments, we set a number of loci and alleles in genotype *LA* > *P_e_* to ensure that alternative genetic variants encoded the same phenotypic element. This made the genotype–phenotype map more evolvable by ensuring some degree of robustness to the mutations. Randomly assigning values to the genes in the initial population gave the probability of the state of an element to be 1, which could be estimated as $p_{rnd}=1-{\left(1-\frac{1}{P_{e}}\right)}^{L}\approx 0.865$ for values used in the simulation (Table 1). The expected number of missed elements in an incomplete functional system $m=C\left(1-p_{rnd}\right)$.

**Figure 2:**
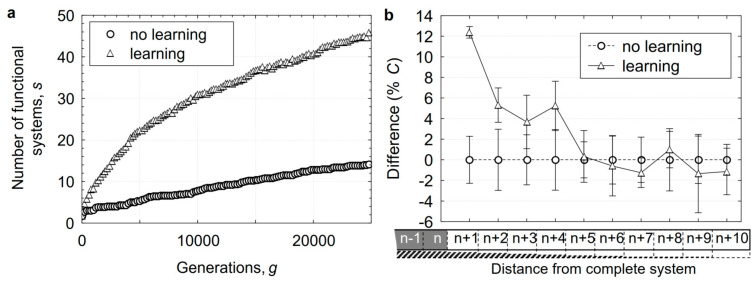
Plasticity accelerates evolution. (**a**) The number of functional systems acquired during evolution increased more rapidly in the populations that exhibited plasticity (triangles) than in the populations that did not (circles). In each generation, the mean of 10 simulations is shown for the individuals with the largest number of functional systems. (**b**) The proportion of inherited elements in systems beyond the last complete system was significantly higher in plastic populations, as shown by 95% confidence limits, than in the non-plastic populations. The difference between the populations decreased with stages more remote from the last complete functional system. For both panels, the number of elements required to generate the functional system *C* = 20, and the mutation rate per gene locus *µ* = 0.0002.

We first studied the evolution of model organisms in populations with and without plasticity. Figure 2a shows how, for a fixed and relatively low mutation rate, the capacity to learn greatly increases the number of sequentially ordered systems that evolve over *n_g_* = 25,000 generations. The rate of the acquisition of systems started to level off because, in this model, the greater the number of systems that were accumulated, the more likely was that the developmental process would be disrupted by mutation. The overall result was robust across a wide range of parameter values (see below). Populations without plasticity gained on average 14.3 systems by the end of 10 runs of the simulation, with the mutation rate per gene locus *µ* = 0.0002. The simulations with plasticity finished with an average of 45.1 systems for the same parameter settings, a rate that is more than three times greater than in the non-plastic population. Therefore, in this model, plasticity promoted a much more rapid genetic evolution of the complex sets of the adaptive systems than the evolution accomplished by mutation alone. This occurred as the previously plastic elements were replaced by inherited elements, and the model organism was able to fill by plasticity the missing elements in subsequent systems (see Figure 1). The proportion of the inherited elements in the systems beyond the last complete functional system was highest in the incomplete systems closest to it (Figure 2b).

The effect of plasticity on the evolution of complex biological structures and behaviours is even more dramatic if the number of generations needed to evolve the same number of functional systems is considered. For the same settings as used in Figure 2a, it took 2458 generations for the plastic populations to accumulate 14.3 systems. This is more than 10 times faster than in the populations that evolved without plasticity.

**Figure 3:**
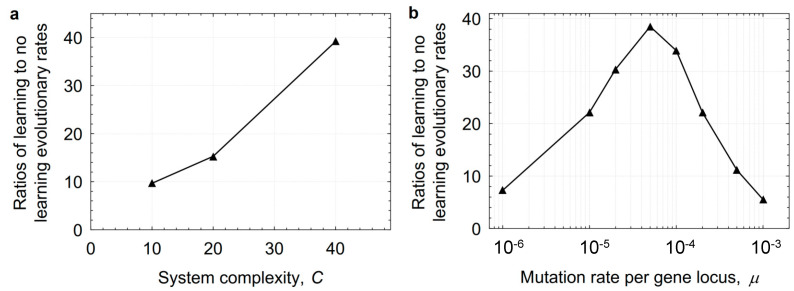
Effects of plasticity on the rates of the evolution of system complexity and mutation. The acquisition rate in the populations that exhibited plasticity relative to those that did not is shown in (**a**) for different values of the parameter *C* that specifies the number of elements required to generate the functional system and provides a measure of the system’s complexity and in (**b**) for different rates of mutation *µ*. The ratios were obtained from the mean of 10 simulations and averaged over the range of mutation rates (μ∈ [10^−6^, 10^−3^]) in (**a**) and over the system’s complexities (*C* = {10;20;40}) in (**b**).

To investigate how the capacity of plasticity to speed up evolution is related to the complexity of the phenotypes required to enable adaptive traits, we conducted simulations by varying the number of elements in each system. The difficulty of acquiring a new system in our model was controlled by the parameter *C*. The value of this parameter determined how many elements were required to make a new system functional. We studied the populations with *C* equal to 10, 20, or 40. The ratio of the generations required to evolve the phenotype with the same number of functional systems in populations with and without plasticity is given in Figure 3a. For both the populations with and without plasticity, the rate of evolution decreased with the task complexity, but on average, the relative potentiating effect of plasticity was four times greater for *C* = 40 than for *C* = 10 (Figure 3a). In the case of the most complex adaptation, plasticity accelerated the accumulation of adaptive systems about 40-fold. On the assumption that the number of functional systems is related to the complexity of biological organisation, these results suggest that plasticity accelerates the evolution of yet more complex organisations. In the model, the acceleration was greater as more elements were added to the systems and the difficulty of completing a terminal system was increased. The chances of the members of the non-plastic populations acquiring solutions to the most complex of problems were much lower than in members of the plastic populations, and, if they did acquire them, required a very long period of evolution.

Next, we studied how the effect of adaptability on evolution depends on the rate of mutation (see Figure 3b). With an increasing mutation rate from *µ* = 10^−6^ to 10^−3^, the acceleration of evolution by plasticity first grew to a maximum at *µ* = 10^−4^ and then decreased. For high mutation rates, acceleration decreased due to the disruption of already established adaptive elements. For the lower rates of mutation, the effect of the mutation rate on the difference between the plastic and non-plastic populations is explained by the likelihood that a plastic element will be replaced by an inherited one.

**Figure 4:**
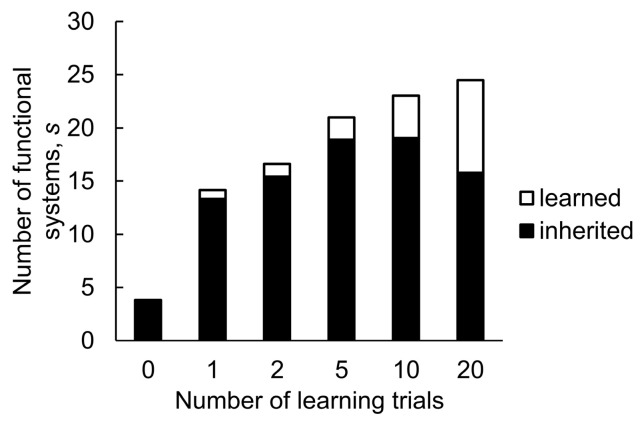
The number of functional systems after 25,000 generations with varying amount of learning specified by the parameter *t*. The data were obtained from the mean of 10 simulations and averaged over the range of mutation rates (*µ* = 10^−5^–10^−3^) with the complexity *C* = 40.

Finally, we studied how the rate of evolution changed with an increasing number of learning trials in populations capable of learning (Figure 4). Even one learning trial was sufficient to significantly accelerate the accumulation of adaptive phenotypic modifications. Populations with two or five learning trials evolved faster than populations with one trial. A higher lifetime capacity for adaptation results in the evolution of more complex phenotypes, but the most plastic population in our experiments developed fewer inherited functional systems, demonstrating the shadowing effect of plasticity.

## 4. Discussion

Inasmuch as it has been taken seriously, the adaptability driver has usually been taken as providing a mechanism for the slow accretion of spontaneously expressed phenotypic elements in the course of evolution [38]. Emphasis has been placed on how particular behaviour patterns initially acquired by learning could be expressed spontaneously without learning in the course of subsequent evolution [10]. Recent developments initiated by the work of Hinton and Nowlan [32] have shifted the focus to other issues, such as the way in which plasticity can accelerate the rate at which challenges set by the environment can be met [27,28,31,57,58], the advantages of plasticity in a changing environment [29,33,36,58,59,60,61,62], and the conditions in which plasticity might slow down evolution [27,31]. Our study complements such proposals about the role of plasticity in the evolutionary processes. However, this study adds a much more important feature to the general discussion of developmental plasticity and evolution. By incorporating Pyotr Anokhin’s [55] concept of partially overlapping functional systems, the simulations indicate how plasticity could have facilitated rapid evolutionary change. From the perspective of a single ecological challenge requiring just one functional system, our model is similar to a classical single-peaked landscape simulation [32]. However, the main highlight of our model is its operation in a complex ecological landscape in which many challenges face the organism [63]. It allowed us to examine the role of plasticity in the evolution of multiple partially overlapping functional systems. We were not concerned here with how the capacity to learn from others affects genetic change, which was the central focus of Boyd and Richerson and their colleagues [64].

Evolutionary theory has provided elegant explanations for the ways in which complexity might be gradually elaborated. The many steps from simple light detectors to complex vertebrate eyes have been well-described [65,66]. Major transitions in evolution have been explained in terms of the changes in genetic regulation early in development [67], and these explanations have been offered for the explosion of variety seen in the Cambrian [68]. Our study suggests another way in which the process of rapid evolution might have been driven, particularly in more complex animals. The model demonstrates how mutations, each of which produces small variations, can be accumulated under the guidance of plasticity to create a substantial adaptive change of the phenotype. The model explains how rapid evolutionary change can be squared with maintaining the overall functionality of the organism [69].

If we are correct, the role of plasticity would have become more and more important as phenotypes increased in complexity. Furthermore, if plasticity had not been possible until a certain level of complexity had evolved, then a sharp increase would occur in the rate of evolution at that point. By varying the amount of plasticity in the model, we found that it affected the speed of evolution in a nonlinear manner. Some theorists have argued that plasticity could dampen the rate of evolution e.g., [70,71]. Their proposal was that, with every individual in the population coping plastically with an environmental challenge, natural selection would have had no variation on which to act. In some cases, this might well have been true in the short run. However, if operating plastic mechanisms involved time and energy costs, then the individuals that expressed the adaptation spontaneously would readily invade the population, and the dampening effect of plasticity on evolutionary rate would be lost.

## 5. Conclusions

In general, our simulations suggest that, thanks to an ability to cope with complex environmental challenges by adding to the elements of existing functional systems, plasticity opens up ecological niches previously unavailable to an organism. These plastic changes could include the creative effects of play [72]. The beneficial effects would inevitably lead to the subsequent evolution of morphological, physiological, and biochemical adaptations in those previously unoccupied niches. Where an environmental challenge involved greater processing capacity by the brain, this organ too would be expected to evolve with greater rapidity. On the assumption that a bigger brain ensures a greater learning capacity, the rate of evolution should correlate positively with the relative brain size. This expectation is given some support by this study, suggesting that the taxonomic groups that evolve most rapidly have the biggest brains relative to body size [51]. This expectation is also supported by the correlation between behavioural innovation and brain size reported for birds [52] and primates [73]. As the beneficial consequences of the completed functional systems became available to it, the animal would have then been able to acquire by learning adaptations to new challenges set by the environment. We conclude, therefore, that each individual’s plasticity provided an evolutionary ratchet, and this provided direction toward ever-greater complexity.

## Figures and Tables

**Table 1:** Definition of parameters, variables, and corresponding values used in the simulation.

Parameter	Description	Value(s)
*N*	population size	500
*n_g_*	number of generations in simulated evolution	25,000
*L*	number of loci in genotype	4000
*A*	number of alleles per gene locus	5
*µ*	probability of point mutation	10^−6^–10^−3^
*f*	number of functional systems per individual in the simulation	*P_e_*/*C*
*P_e_*	number of phenotypic elements per individual	2000
*C*	number of phenotypic elements required to acquire a new functional system (i.e., the complexity of new system acquisition)	10, 20, 40
*p_e_*	probability of filling in missing phenotypic elements in the terminal system during learning trial	0.5
*t*	number of learning trials	0, 1, 5, 10
**e** *^i^*	a string of zeroes and ones that describes the composition of the *i*^th^ functional system	
*m*	the number of missed elements in an incomplete functional system	

## Data Availability

The code for this paper is available at https://github.com/burtsev/EvoLearn (accessed on 19 August 2024).
